# Recommendations for Improving Identification and Quantification in Non-Targeted, GC-MS-Based Metabolomic Profiling of Human Plasma

**DOI:** 10.3390/metabo7030045

**Published:** 2017-08-25

**Authors:** Hanghang Wang, Michael J. Muehlbauer, Sara K. O’Neal, Christopher B. Newgard, Elizabeth R. Hauser, James R. Bain, Svati H. Shah

**Affiliations:** 1 Duke Molecular Physiology Institute, Duke University School of Medicine, Durham, NC 27701, USA; hanghang.wang@duke.edu (H.W.); michael.muehlbauer@duke.edu (M.J.M.); sara.oneal@duke.edu (S.K.O’N.); chris.newgard@duke.edu (C.B.N.); elizabeth.hauser@duke.edu (E.R.H); 2 Department of Surgery, Duke University School of Medicine, Durham, NC 27710, USA; 3 Division of Cardiology, Department of Medicine, Duke University School of Medicine, Durham, NC 27710, USA

**Keywords:** metabolomic profiling, GC-MS, human plasma

## Abstract

The field of metabolomics as applied to human disease and health is rapidly expanding. In recent efforts of metabolomics research, greater emphasis has been placed on quality control and method validation. In this study, we report an experience with quality control and a practical application of method validation. Specifically, we sought to identify and modify steps in gas chromatography-mass spectrometry (GC-MS)-based, non-targeted metabolomic profiling of human plasma that could influence metabolite identification and quantification. Our experimental design included two studies: (1) a limiting-dilution study, which investigated the effects of dilution on analyte identification and quantification; and (2) a concentration-specific study, which compared the optimal plasma extract volume established in the first study with the volume used in the current institutional protocol. We confirmed that contaminants, concentration, repeatability and intermediate precision are major factors influencing metabolite identification and quantification. In addition, we established methods for improved metabolite identification and quantification, which were summarized to provide recommendations for experimental design of GC-MS-based non-targeted profiling of human plasma.

## 1. Introduction

High-throughput molecular profiling is being increasingly used in large numbers of human samples to identify novel biomarkers and mechanisms of health and disease. Metabolomics, a subfield in molecular profiling, investigates the metabolome, the total quantitative collection of small-molecule metabolites in biofluids such as plasma [1,2], in an identified and quantified manner [3]. Since metabolomic changes are downstream of alterations at the genomic, transcriptomic and proteomic level [4], metabolomics is particularly suited for identification of biomarkers, examination of molecular physiology, and investigation of genetic and environmental modifications [5]. Commonly utilized technologies in metabolomics include liquid or gas chromatography (LC and GC) coupled to mass spectrometry (MS), capillary electrophoresis (CE), and nuclear magnetic resonance (NMR) spectroscopy [6].

Two general approaches exist in metabolomic profiling: targeted and non-targeted. The targeted approach identifies and quantifies select known metabolites, usually via isotope-labeled internal standards; the non-targeted approach aims to profile as many metabolites as possible, the identities of which are not established prior to analysis. The main advantage of the non-targeted approach is a broader coverage of the metabolome with opportunities for discovering novel pathways [7]. However, the non-targeted approach comes with inherent challenges surrounding metabolite identification and quantification, the first step of metabolomic profiling that directly impacts further biological insight. These challenges arise from both the complexity of biofluids with a wide range of compound classes and metabolite abundance, and intrinsic limitations of available analytical techniques [8,9]. For example, unknowns or analytes with no chemical identification discovered in metabolomic profiling studies frequently exceed the number of known metabolites with positive or putative identification by 2–3 times [10,11]. In recent years, with the advancements in metabolomic profiling approaches, more insights have been gained into the human blood metabolome [12,13]. Comprehensive databases for the human blood metabolome such as the Human Metabolome Database (http://www.hmdb.ca) have also been constructed to improve the identification of metabolites and characterization of metabolic pathways. Systematic metabolite identification and quantification in non-targeted metabolomic profiling have resulted in the discovery of novel disease biomarkers and pathways [14], while overlooking these key steps prior to drawing biological inferences has led to early pitfalls [15]. 

Compared to the targeted approach, non-targeted metabolomic profiling is also associated with greater difficulties in quality control and method validation where common parameters considered in method validation of targeted analysis such as accuracy or trueness cannot be adapted easily [16]. Efforts to overcome these difficulties, both at the experimental and computational level, have become a focus of metabolomic research [17,18,19,20,21,22,23]. These efforts include research on standardizing the experimental protocols [19,24,25], strategies for incorporating quality controls [18,26,27,28,29], and recommendations for employing statistics in the experiment design [30]. These efforts have also resulted in the formation of many working groups and data repositories (e.g., Metabolomics Workbench [31]) for standardization [17,32]. Currently, more research is still needed in the practical applications of quality control and method validation. For example, while chemical contamination has been suggested to interfere with metabolomic profiling [14], no previous study has investigated the effect of contaminants on metabolite identification and quantification systematically. As a result, recommendations for experimental design frequently include the incorporation of blanks containing identical reagents as biological samples [7,18,27], but few recommendations exist concerning ways to process and utilize blank data. The effect of concentration on metabolite identification has also been reported, where increased concentration resulted in higher numbers of identified components [33], but most of these studies were restricted to standard solutions [33] or a subset of isotope-labeled metabolites [34]. While some studies have advocated the use of linearity and repeatability and intermediate precision in quality control samples to monitor analytical performance [7,19,27], few have investigated their effects on metabolite quantification in complex biological samples or provided practical guidelines for improvement. 

In this study, we sought to identify and modify steps in non-targeted metabolomic profiling of human plasma that could influence metabolite identification and quantification. We performed non-targeted metabolomic profiling using gas chromatography-mass spectrometry (GC-MS), due to its broad coverage, high sensitivity, and reproducibility [2,35,36]. Our hypothesis is that contaminants, concentration, and repeatability and intermediate precision are major factors influencing metabolite identification and quantification. In addition to developing methods for improved identification and quantification, we hope to provide recommendations for experimental design in GC-MS-based non-targeted metabolomic profiling of human plasma.

## 2. Results

Our experimental design included two studies: (1) the limiting-dilution study, which investigated the effects of dilution on analyte identification and quantification, and (2) the concentration-specific study, which compared the optimal concentration established in the first study with the standard volume used in the current institutional protocol [37]. For both studies, aliquots of human plasma were deproteinated with methanol, dried, methoxymated, trimethylsilylated, and run on a 6890N GC/5975 Inert MS (Agilent Technologies, Santa Clara, CA, USA). 

Results from all aliquots were included in the analysis. A total of 320 analytes were detected in the limiting-dilution study, consisting of 183 known analytes and 137 unknowns. After excluding 29 analytes present in less than 20% of non-blanks (known: 24, unknown: 5), 291 analytes (known: 159, unknown: 132) were included in further analysis. 

### 2.1. Selectivity: Contaminant Profile

**Figure 1:**
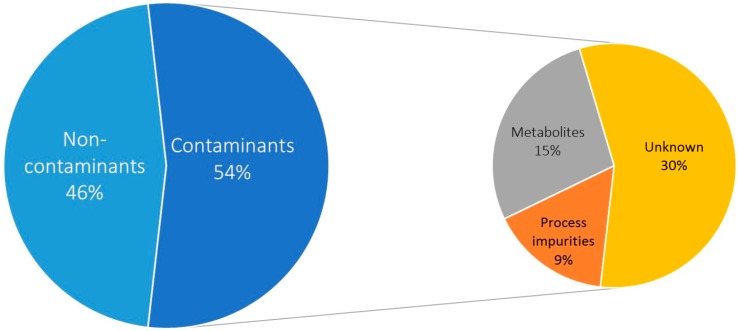
Contaminants represent 54% of all analytes detected. Classes of contaminants: process impurities (e.g., silicone oils and alkane hydrocarbons) present in blanks or discovered after manual curation (25), metabolites present in blanks (43), and unknowns present in blanks (88).

The selectivity of an analytical method is defined as the ability to quantify the analytes accurately in the presence of interferences, such as process impurities and chemical contamination [18,38]. Examination of the contaminant profile can prevent false-positive discoveries and increase the selectivity of non-targeted metabolomic profiling. In the limiting-dilution study, 156 out of the 291 (53.6%) profiled analytes were present in at least one blank (148) or annotated as non-metabolites after manual curation (8). These analytes were characterized as contaminants, and were further classified into definite (present in greater than or equal to 20% or 6 blanks or annotated as a non-metabolite after manual curation, 123) or potential contaminants (present in greater than or equal to 1 but less than 6 blanks, 33). Classes of these contaminants (Figure 1) include process impurities (e.g., silicone oils and alkane hydrocarbons) present in blanks or discovered after manual curation (Supplemental Table S1), metabolites present in blanks (Table 1), and unknowns present in blanks. The majority of unknown (66.7%, 88) and 42.8% (68) of known analytes were contaminants. 

Five contaminants exhibited positive run-order effects (Spearman’s rho greater than 0.5, *p*-value less than 0.05), including four unknowns and one equipment component; 16 contaminants exhibited negative run-order effects (Spearman’s rho less than −0.5, *p*-value less than 0.05), including 1 equipment component, 4 metabolites and 11 unknowns (Supplemental Figure S1). 

The majority (49, 72.1%) of the 68 known contaminants (41 definite, 8 potential) from the limiting-dilution study were reproducible in the concentration-specific study. The majority of non-reproducible known contaminants were metabolites (16) undetected in blanks in the concentration-specific study. The concentration-specific study also produced 3 new contaminants. Additionally, 12 of the 74 (16.2%) unknown definite contaminants, as characterized by a match from the auxiliary library of unknowns, were reproducible. These results were used to establish a contaminant repository consisting of highly reproducible and potential contaminants for reference in future studies. 

Definite non-metabolite contaminants (equipment components and unknowns, 98) and reagent derivatives (EDTA, MSTFA and pyridine derivatives, 5) were excluded from further analysis. Potential contaminants were included after background adjustment by subtracting the mean batch-specific blank level from the analyte level. Five potential contaminants with unadjusted levels lower than the background were excluded. Combined with noncontaminants, 183 analytes remained as features to describe potentially authentic metabolites. Known analytes identified in the NIST SRM1950 plasma were consistent with those reported in previous publications [28]. The identities of these analytes, together with analytes identified in the volunteer plasma, are listed in Supplemental Table S2.

### 2.2. Linearity: Signal-Concentration Relationship

Linearity refers to the ability to obtain measured analytical signals directly proportional to the concentration of analytes [39]. Linearity is a multifactorial problem affected by ionization efficiency of the analyte, ion transport from the ion source to the mass analyzer, and linear response of the detector. Assessment of the linearity of this signal-concentration relationship provides validation to simultaneous measurement of multiple metabolite concentrations in non-targeted metabolomic profiling [19]. In the limiting-dilution study, the linear regression model was deemed appropriate by F-test in 112 (61.2%) analytes, including 74 known analytes and 38 unknowns. After excluding 16 definite or potential contaminants, 55 analytes exhibiting lack of fit for the linear model were refitted with sigmoid curves using logistic regression models, as well as polynomial models (quadratic, cubic or 4th order), to test the hypothesis that saturation of the chromatography column is responsible for the lack of fit. F-test revealed that sigmoid curves were appropriate for 26 analytes and polynomial models were appropriate for 18 analytes in this subgroup, confirming the effects of saturation. 

**Figure 2:**
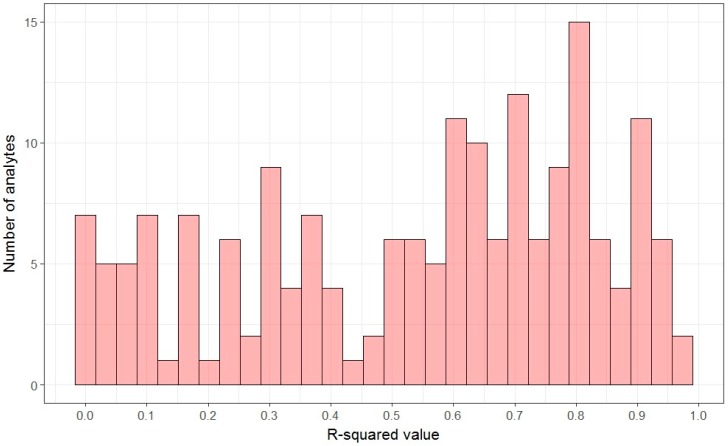
Distribution of R^2^ value for all analytes. Approximately half of analytes (47.9%, 23) with low linearity (R^2^ less than 0.5) were definite or potential contaminants.

For the 112 analytes where the use of the linear regression model was appropriate, the adjusted R^2^ was used to assess the degree of linearity (Figure 2). Approximately half of analytes (47.9%, 23) with low linearity (R^2^ less than 0.5) were definite or potential contaminants. Known analytes had a significantly higher linearity than unknown analytes (*p* = 0.01, Table 2). Examination of the estimated parameter $\beta_{1}$ revealed that all except one analyte, a potential contaminant, had positive slopes. 

### 2.3. Linear Dynamic Range

The linear dynamic range can be used to determine the optimal range for analyte detection. Outside the linear dynamic range, estimation of the analyte concentration becomes uncertain and may deviate significantly from the actual value [39]. In the limiting-dilution study, the majority (90.5%) of analytes’ linear dynamic range (LDR) was between concentrations of 4.98 × 10^−9^ and 7.48 × 10^−9^ (*v*/*v*, corresponding to a plasma extract volume of 100–150 µL) or 7.48 × 10^−9^ and 9.97 × 10^−9^ (corresponding to a plasma extract volume of 150–200 µL, Table 3). Only one analyte’s LDR was above 1.50 × 10^−8^ (plasma extract volume 300 µL). Using this information, the concentration of 7.48 × 10^−9^ (plasma extract volume 150 µL) was determined optimal. 

### 2.4. Repeatability and Intermediate Precision

**Figure 3:**
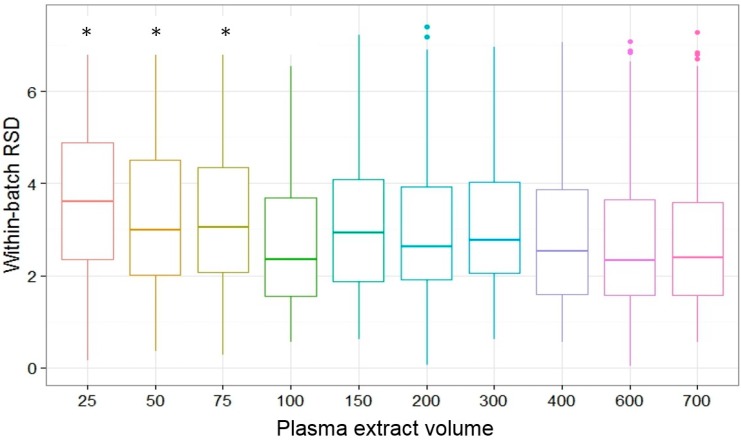
Boxplot of repeatability (within-batch relative standard deviation or RSD) by plasma extract volume. The horizontal lines represent the median and the lower and upper hinges correspond to the 25th and 75th percentiles. The asterisks * denote RSD that was significantly different in post-hoc pairwise comparisons using the Conover’s test for multiple comparisons.

Since all plasma extracts used in this study were obtained from one sample (single blood draw from one individual), biological variability was minimized. Therefore, repeatability and intermediate precision in this study reflected mainly of process variability in sample preparation and instrument variability; each plasma extract aliquot served as quality control. Median within-batch RSD for all analytes was significantly higher at low plasma extract volumes than at high volumes (Figure 3, Kruskal-Wallis rank sum test, *p*-value less than 0.001). Post-hoc pairwise comparisons using the Conover’s test for multiple comparisons revealed that this difference was significant for the lowest three volumes (25, 50 and 75 µL) and no longer significant starting at 100 µL.

Averaged across all plasma extract volumes, within-batch RSD was significantly higher in definite and possible contaminants (median = 3.42, 25th/75th: 2.48/5.13) than non-contaminants (median = 3.06, 25th/75th: 2.33/3.92, Wilcoxon rank sum test, *p*-value = 0.04). Analytes with low linearity also had significantly higher within-batch RSD (median = 4.50, 25th/75th: 3.15, 4.93) than analytes with high linearity (median = 2.33, 25th/75th: 1.73/2.83, Wilcoxon rank sum test, *p*-value less than 0.001). There was no significant difference in within-batch RSD for known analytes vs. unknowns (Wilcoxon rank sum test, *p*-value = 0.22).

**Figure 4:**
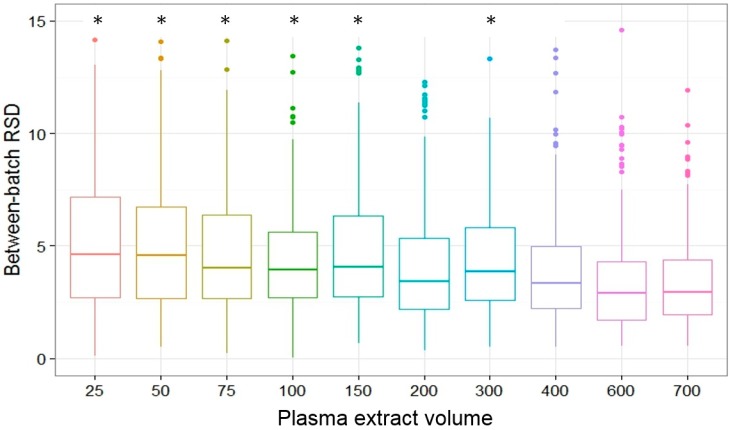
Boxplot of intermediate precision (between-batch relative standard deviation or RSD) by plasma extract volume. The horizontal lines represent the median and the lower and upper hinges correspond to the 25th and 75th percentiles. The asterisks * denote RSD that was significantly different in post-hoc pairwise comparisons using the Conover’s test for multiple comparisons.

The median between-batch RSD for all analytes was significantly higher at lower volumes than at high volumes (Figure 4, Kruskal-Wallis rank sum test, *p*-value less than 0.001). Post-hoc pairwise comparisons revealed that this difference was significant for all volumes below 400 µL. Between-batch RSD was larger than within-batch RSD for 141 (76.2%) analytes.

Averaged across all plasma extract volumes, between-batch RSD was significantly higher in definite and possible contaminants (median = 5.22, 25th/75th: 3.82/7.63) than non-contaminants (median = 3.72, 25th/75th: 2.81/4.75, Wilcoxon rank sum test, *p*-value less than 0.001). Analytes with low linearity also had significantly higher within-batch RSD (median = 6.46, 25th/75th: 4.64/7.95) than analytes with high linearity (median = 2.86, 25th/75th: 2.33/3.42, Wilcoxon rank sum test, *p*-value less than 0.001). There was no significant difference in within-batch RSD for known analytes vs. unknowns (Wilcoxon rank sum test, *p*-value = 0.18). 

An analysis-of-variance (ANOVA) test comparing a linear regression model with the addition of a batch variable and the basic model revealed that 173 (93.5%) analytes exhibited significant intermediate precision to warrant the inclusion of a batch variable in the analysis.

### 2.5. Concentration-Specific Study

After exclusion of contaminants, 133 known analytes detected in the concentration-specific study were compared to the limiting-dilution study. The majority of these analytes (117, 88.0%) were detected previously in the limiting-dilution study. Analytes not previously detected (16) were considered non-reproducible and excluded from the concentration comparisons. 

Comparison of analyte detection at plasma extract volume 150 vs. 700 µL (plasma concentration 7.48 × 10^−9^ vs. 3.49 × 10^−8^) revealed 7 known analytes and 13 unknowns that were detected inconsistently (less than 20%) at 150 µL and consistently (greater than 50%) at 700 µL. All seven known analytes except one were of low to moderate linearity (adjusted R^2^ less than 0.7) in the limiting-dilution study.

## 3. Discussion

In this study, we investigated the steps in GC-MS-based non-targeted metabolomic profiling of human plasma that could influence metabolite identification and quantification. We tested and confirmed that contaminants, concentration, and repeatability and intermediate precision are major factors influencing the identification and quantification of metabolites. The findings of this study lead to recommendations for experimental design in GC-MS-based non-targeted metabolomic profiling of human plasma. 

Through methodical inclusion and systematic analysis of blanks, we discovered that the majority of unknowns and close to half of known analytes detected were contaminants. This result highlights the importance of including blanks in GC-MS-based non-targeted metabolomic profiling, a step that is not universally incorporated in practice currently. While the majority of contaminants were equipment components, unknowns, or reagent derivatives, 19% were metabolites with levels above the detection limit but below true biological levels. These metabolite contaminants consist of a wide range of metabolites, such as amino acids, carbohydrates, fatty acids, lipids and organic acids. The most likely sources of metabolite contaminants are the polypropylene tubes used in sample preparation, with oils used as extrusion aids or mould-release agents. Our results provide direct evidence that contaminants could share similar chemical and physical properties to true metabolites, as proposed previously by Dunn et al. [27]. Without background correction, these metabolite impurities could affect the selectivity of metabolite quantification by providing false positive signals. While inclusion of blanks may increase the cost of metabolomic assays, the additional information gained in both metabolite identification and quantification warrants investigators considering routinely including them in study designs. In addition to improving selectivity, our results also demonstrated that using blanks could provide insight into the nature of unknowns and significantly narrow their search space. Unknowns are often considered spurious peaks from reagent contaminants, chemical artifacts during derivatization or deconvolution artifacts as opposed to true metabolites, and most current studies exclude all unknowns routinely from further analysis. While some studies have reported the number of unknowns [40], few have reported their characteristics or distribution. In this study, we discovered that while the majority of unknowns were contaminants, some were absent in blanks, results that were reproducible in the second study. By including reproducible unknowns in metabolomic profiling, the statistical power could be increased, potentially leading to the discovery of novel biomarkers and pathways. 

Comparison of the limiting-dilution and concentration-specific study showed that the contaminant profile is highly reproducible. This result prompted us to establish a contaminant repository consisting of highly reproducible and potential contaminants for reference in future studies. 

Few previous studies have explored the signal-concentration relationship in complex biological samples such as human plasma [34]. Our study utilized analytical replicates to examine the appropriateness of a linear model through comparing the pure error variability and variability from lack of fit. In our study, the signal-concentration relationship was linear for only 61.2% of analytes. Potential explanations for nonlinearity include contaminant effect and saturation effect. Contaminant effect arises from the metabolite impurities present in equipment and reagents that could affect the samples differently. At lower concentrations, false positive signals may arise from these impurities, thus affecting metabolite quantification. Conversely, as concentration increases beyond a certain threshold, the chromatography column may become saturated, resulting in peak broadening, decreased sensitivity and poor quantification. In this study, we examined saturation effect using sigmoid and polynomial models as alternatives to the linear regression model. Our results showed that saturation effect could explain close to half of the nonlinearity. 

Our results showed that known analytes had significantly higher linearity than unknowns. This is likely because many unknowns may be spurious peaks arising from deconvolution artifacts or impurities. The classes of metabolites represented by linear analytes are diverse, suggesting that the functional group is not the only factor that affects linearity. Previous studies have advocated using dilution in quality control samples of metabolomic profiling to generate a list of highly linear “targets” that can be used for further method validation [7]. These known analytes showing high linearity in this study were used to construct a list of targets that we will use for performance monitoring in the future; the unknowns showing high linearity were added to our institutional library as potential metabolites of biological importance. 

By examining the linear dynamic range for all analytes, we determined that the optimal concentration for quantification was 7.48 × 10^−9^ for the majority of analytes, corresponding to a plasma extract volume of 150 µL. The optimal protocol established for sample preparation and derivatization (SOP) can be found at: http://dmpi.duke.edu/files/dmpi_gc-ms_protocol.pdf. The subsequent concentration-specific study confirmed that by decreasing the plasma extract volume from 700 to 150 µL (concentration from 3.49 × 10^−8^ to 7.48 × 10^−9^), only a few low abundant, low linear metabolites and unknowns were less consistently detected. One of the main challenges in metabolomic profiling is the trade-off between detection and quantification. Using higher plasma volumes may increase the detection rate of low abundant analytes. However, at higher volumes, peaks for highly abundant analytes may become saturated, resulting in decreased accuracy in quantification. In the application of metabolomic profiling to human diseases, quantification of most analytes may be more important than detection of low abundant analytes, especially when the goal is to differentiate as many metabolite levels between cases and controls as possible. Conversely, for studies on samples with low abundant metabolites (e.g., neonates), using a higher plasma volume and thus metabolite concentration may be required to achieve improved identification and quantification. Of note, the optimal plasma volume established in this study may not be generalizable to other studies using different analytical instruments and experimental conditions. Therefore, we recommend establishing the linear dynamic range specific to individual instruments prior to initiating large-scale non-targeted metabolomic profiling studies. 

In this study, repeatability was greatest at the lowest three volumes. This result is consistent with previous reports [34]. Sources of repeatability include variability in sample preparation and data acquisition. Specifically, contaminants affected repeatability significantly, as evidenced by higher within-batch RSD in contaminants than non-contaminants. The fact that within-batch RSD did not differ in known analytes compared to unknowns suggests that repeatability is intrinsic to the experimental process, rather than analyte-specific. The overall low within-batch RSD confirms that the method is highly reproducible, and meets the requirements similar to targeted methods. 

Intermediate precision was higher than repeatability for the majority of analytes in this study. Sources of intermediate precision are similar to repeatability and include variability in sample preparation and data acquisition. In addition, since different batches were performed on different days, change in sensitivity over time may also contribute to intermediate precision as sample components aggregate in the GC injector or electrospray ion source [27]. While inter-experiment RSD was below 10% for the majority of analytes at all concentrations, the significant batch effect on quantification for most analytes suggests that batch controls should be included routinely in reporting and analysis of metabolomic profiling. 

Broad-scan, non-targeted GC/MS metabolomics is useful for examining small compounds in plasma whose concentrations range from low micromolar to millimolar. However, GC has numerous limitations, including the need to extract and derivatize analytes to render them sufficiently nonpolar for GC. GC is poorly suited for some compounds, including those that are highly volatile and elute in the solvent front, as well as thermolabile or highly polar metabolites, such as quaternary amines, guanidino compounds, internal zwitterions, and molecules with phosphodiester bonds. Protocols and instruments vary widely. In assays for the hundred-plus plasma metabolites that are readily accessible by GC/MS, optimization experiments are essential during development of a stable analytic platform.

## 4. Materials and Methods

**Figure 5:**
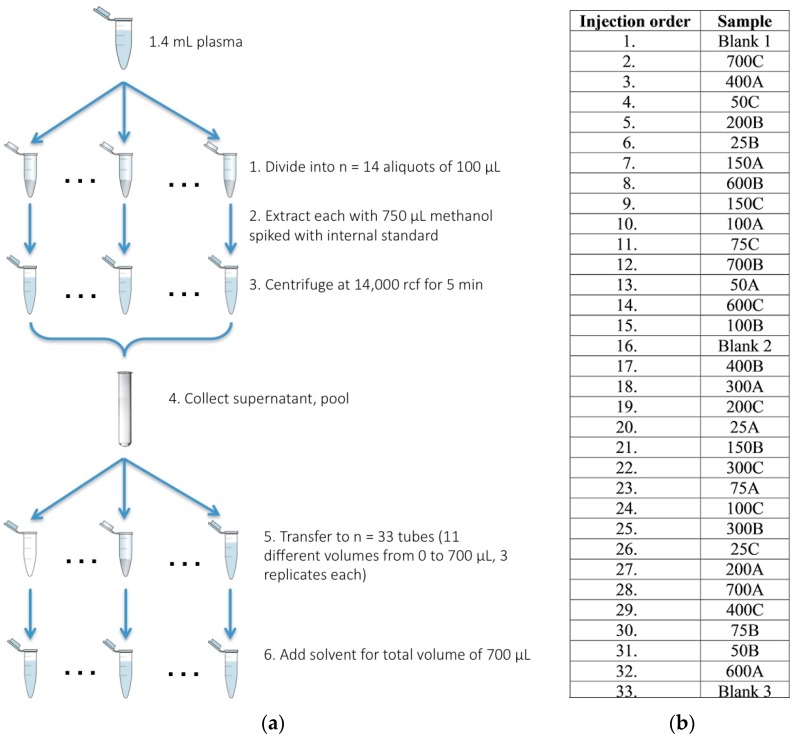
(**a**) Schematic of the sample preparation steps for the limiting dilution study; (**b**) an example of the injection order of the plasma extract aliquots. Aliquots were analysed in a randomized order to minimize biases in sample preparation and data acquisition. Blanks containing the reagents only were included in at the beginning, middle, and end of each run. The concentration-specific study used a similar protocol except for different plasma extract volumes (0, 150 and 700 µL only).

Our experimental design included two studies: (1) the limiting-dilution study (Figure 5), which investigated the effects of dilution on analyte identification and quantification, and (2) the concentration-specific study, which compared the optimal concentration established in the first study (7.48 × 10^−9^, corresponding to a plasma extract volume of 150 µL) with the standard volume used in the current institutional protocol [37] (3.49 × 10^−8^, corresponding to a plasma extract volume of 700 µL).

### 4.1. Sample Acquisition, Preparation, and Derivatization

Both studies utilized a single EDTA-anticoagulated blood sample obtained from one healthy volunteer after 10 h of fasting. The blood sample was collected at the beginning of the limiting-dilution study and plasma was extracted after centrifugation. The plasma sample was then separated into 1.2 mL aliquots and stored at −80 °C prior to sample preparation. 

The limiting-dilution study was divided into 10 batches with identical experimental design (Figure 5) spanning 16 consecutive days, while the concentration-specific study was conducted within a two-day period. For both studies, plasma aliquots (100 µL each) were first extracted with 750 µL methanol spiked with a retention-time-lock internal standard of 6.25 mg/L perdeuterated myristic acid (C14:0-D27-TMS) to remove proteins. Following centrifugation at 2081× *g* for 5 min at room temperature, the supernatants were pooled into a 10 mL glass tube. Varying amounts of the pooled methanolic extract were then dispensed into new microcentrifuge tubes, and ballasted with 7.5:1 MeOH/H_2_O (*v*/*v*) for a total volume of 700 µL. The limiting-dilution and concentration-specific study differed in the volumes of pooled methanolic extract used, corresponding to different plasma concentrations. For the limiting-dilution study, each batch consisted of 33 aliquots with 11 different plasma extract volumes (0–700 µL), corresponding to 11 plasma concentrations repeated three times (Table 4). The concentration-specific study consisted of one batch of 32 aliquots: 15 replicates for each of the two concentrations 7.48 × 10^−9^ and 3.49 × 10^−8^ (corresponding to plasma extract volumes of 150 µL and 700 µL, respectively) and two blanks (reagents only). For both studies, each aliquot of methanolic extract ballasted with MeOH/H_2_O was dried with a SpeedVac SPD111V sample concentrator (Thermo Fisher Scientific, Asheville, NC, USA) for 5 h, followed by the addition of 100 µL ethyl acetate as an azeotropic drying agent, and another 45 min of SpeedVac drying. The dried plasma extracts were derivatized with 25 µL of 18 mg/mL methoxyamine hydrochloride in pyridine at 50 °C for 30 min, followed by trimethylsilylation with 75 µL of *N*-methyl-*N*-(trimethylsilyl)trifluoroacetamide (MSTFA) at 50 °C for 30 min.

### 4.2. GC-MS Analysis

The derivatized aliquots were analyzed with a 6890N GC-5975 Inert MS (Agilent Technologies, Santa Clara, CA, USA) using previously described methods [37]. A high-volume, ProSep inlet (liner dimensions 2 × 6.0 × 243 mm, Patent No: US 6,484,560 B1, Apex Technologies, Inc., Edison, NJ, USA) [37] was used to allow for programmed-temperature vaporization and diversion of the heavy contaminants away from the GC-MS. Volumes of 5 µL were injected into a DB5-MS capillary column (two 15 m × 250 µm × 0.25 µm; J & W Scientific, Folson, CA, USA connected in series by a microfluidic flow controller, Agilent Technologies, Santa Clara, CA, USA) in 25:1 split mode. The split ratio was determined empirically in prior experiments. Initial inlet pressures were adjusted empirically to achieve a retention time of 16.727 min for the internal standard. Helium was used as the carrier gas, and the pressure was programmed with helium flow at a constant rate of 2.0 mL/min. The initial GC oven temperature was 60 °C, and the temperature was increased at a rate of 10 °C /min to a final temperature of 325 °C. At the end of each run, both the inlet and the oven were held at 325 °C for a “bake-out” to minimize carryover. During this “bake-out”, the upstream GC column was back-flushed via the mid-column microfluidic splitter, while the inlet was purged with high-flow helium at 50 mL/min. Positive ions were generated with conventional electron ionization (EI) at 70 eV; detection was achieved using a full scan mode from 600 to 50 *m*/*z*. Aliquots were run in a randomized order to ensure that the orders of sample preparation and data acquisition did not introduce biases (Figure 5). Method blanks containing the reagents only were processed following the same procedure as the biological aliquots and included at the beginning, middle, and end of each run. 

Instrument maintenance was performed after every week of analysis, and included cleaning the ionization source components, tuning the mass spectrometry analyzer, and changing the GC liner. After instrument maintenance, injections of the same volunteer plasma were performed prior to continuing the study to compare the retention times, analyte detection, and peak shapes to ensure consistency. 

### 4.3. Metabolite Identification and Quantification

GC-MS data were first deconvoluted with AMDIS (build 140.24, version 2.72, National Institute of Standards and Technology, Gaithersburg, MD, USA), with the following settings, which experience has shown to be suitable: component width 12 scans; exclusions of the total-ion chromatogram and *m*/*z* 73, 74, 75, 147, 148, and 149; adjacent peak subtraction-none; resolution-medium; sensitivity-high; and shape requirements-low. Peak annotation was achieved using our institutional library. The institutional library consists of the Fiehn RTL spectral library [41] with additions established using purified standard compounds in the DMPI metabolomics laboratory and spectra from the Golm Metabolome Database [42] and similar public spectral libraries. Metabolite identification was based on retention index and spectral match scores. Identified (known) analytes with reverse scores greater than or equal to 75 were included in further analysis. Unidentified (unknown) analytes were catalogued using an auxiliary library of spectra corresponding to unidentified peaks that were conserved across samples. These were categorized according to retention index and the dominant *m*/*z* spectral fragment. Retention indices were assigned by a quadratic equation defining the retention index (RI) as a function of retention time (RT), derived from injections of a ladder of fatty acid methyl esters, or FAMES, where RI = 2.246 × RT^2^ + (21.61 × RT) + 507.9, with the RIs of FAMES defined as 800 for methyl octanoate, 900 for methyl nonanoate, and so on. Analyte levels were reported as the log-base-2 transformed values of integrated peak areas. Analytes detected in less than 20% of non-blanks were excluded from further analysis. 

To validate findings in these two studies, a third study was conducted using paired samples consisting of (1) the volunteer plasma used in the first two studies, and (2) the NIST SRM1950 plasma standard (5 × 1 mL) [28]. These paired samples were prepared and analyzed in three batches using the same methods as the limiting dilution study. Identities of known metabolites detected in the NIST SRM1950 plasma standard were compared to previous reports in the literature [28]. 

### 4.4. Parameters Assessed for Method Development

To test our hypothesis, we examined five parameters previously proposed for bioanalytical method development [7] in the limiting-dilution study: selectivity, linearity, linear dynamic range, and repeatability and intermediate precision. 

Selectivity is defined as the ability to identify and quantify analytes in the presence of potential contaminants such as process impurities, reagent derivatives, and sample carryover [7]. We assessed selectivity through examining analytes detected in blanks, with the assumption that any analyte detectable in greater than or equal to 1 blank is a contaminant. These contaminants may include components from collection tubes and plastic ware, reagent derivatives, and metabolites introduced through the preparation process that mimic the same metabolites present in biological samples. Additional contaminants were discovered by manual curation (examination of the annotation): analytes with non-metabolite annotations (e.g., silicone oils) were also classified as contaminants. All contaminants were further classified into definite (present in greater than or equal to 20%/6 blanks or annotated as a non-metabolite) or potential contaminants (present in greater than or equal to 1 but less than 6 blanks). Run-order effects in blanks were estimated as the Spearman’s correlation coefficient between run order and contaminant levels. 

While accuracy of quantification is not easily achievable in non-targeted metabolomic profiling, linearity, or the ability to obtain signals directly proportional to the concentration of analytes within a given range [7], can be assessed as a measure of quantification. Linearity was commonly assessed using the coefficient of determination, or R^2^, in previous studies [34]. Although convenient, R^2^ is a limited measure in assessing goodness-of-fit of a linear regression model, as non-linear relationships can present with a high R^2^ value. In this study, we took advantage of the analytical replicates in the study design and assessed linearity of the signal-concentration relationship using a linear regression model: $Y_{ij}=\beta_{0}+\beta_{1}X_{j}+\varepsilon_{ij}$, where *Y_ij_* denotes the analyte level for the *i*th aliquot for the *j*th level of *X* (*i* = 1, …, 30; *j* = 1, …, 10), *X_j_* is the log_2_ of plasma extract volume, and *ε_ij_* ~ *^iid^ N* (0, *σ*^2^). The parameters $\beta_{0}$ and $\beta_{1}$ were estimated using the least squares solution. The appropriateness of the linear regression model was examined using residual plots by plotting the residuals against fitted values. Additionally, the F-test for lack of fit was used to test the full model: $Y_{ij}=\mu_{j}+\varepsilon_{ij}$, where *E*[*Y_ij_*] = *μ_j_*, versus the reduced model: $Y_{ij}=\beta_{0}+\beta_{1}X_{j}+\varepsilon_{ij}$. Analytes for which the linear model was deemed appropriate were further assessed using the adjusted coefficient of determination, or adjusted R^2^; analytes exhibiting lack of fit for the linear model were refitted with sigmoid curves using logistic regression or polynomial models. 

Linear dynamic range for each analyte was evaluated using response factors obtained by dividing the analyte levels by their concentrations [38]. The linear range was defined as the range between 0.95 and 1.05 times the average value of the response factors. The optimal concentration was determined as the concentration where the majority of analytes were in their linear dynamic range. 

Repeatability and intermediate precision in analyte quantification were assessed by examining the coefficient of variation or relative standard deviation (RSD). Specifically, repeatability, or within-batch variability, was assessed by examining the RSD for each analyte at each plasma extract volume, averaged across the 10 batches. Intermediate precision, or between-batch variability, was assessed by examining the RSD for each analyte at each plasma extract volume, using the mean analyte levels for each batch. Kruskal-Wallis test with post-hoc pairwise comparisons using the Conover’s test was performed to compare the repeatability and intermediate precision RSD at different volumes. In addition, an analysis-of-variance (ANOVA) test comparing a linear regression model with the addition of a batch variable with the basic linear regression model was used to estimate batch effects. A two-tailed alpha of 0.05 was used. 

The concentration-specific study compared analyte detection, defined as the presence of an analyte above the 20% cut-off, at the optimal concentration established in the first study (7.48 × 10^−9^, corresponding to a plasma extract volume of 150 µL) with the standard used in the current institutional protocol (3.49 × 10^−8^, corresponding to a plasma extract volume of 700 µL). Reproducibility of the contaminant and metabolite profile was also assessed by comparing the results of the limiting-dilution and concentration-specific study.

## 5. Conclusions

Using a limiting-dilution and concentration-specific study, we confirmed that contaminants, repeatability and intermediate precision and concentration are major factors influencing metabolite identification and quantification, and established methods for improved metabolite identification and quantification. These methods are summarized (Table 5) to provide recommendations for experimental design of GC-MS-based non-targeted profiling of human plasma metabolome. 

## Figures and Tables

**Table 1:** Metabolite contaminants detected in blanks by type (definite or potential) and chemical class. Detection rate in blanks varied by metabolite type.

Type	Class	Metabolite	No. of Blanks (%)
Definite	Amino acids	Glycine	6 (20%)
-	Benzene derivatives	Benzoic acid	22 (73.3%)
-	Carbohydrates	Glucose and other aldohexoses	20 (66.7%)
-	-	Sucrose and similar disaccharides	10 (33.3%)
-	Fatty acids	Heptadecanoic acid or Octadecanol	23 (76.7%)
-	-	Myristic acid or Pentadecanol	27 (90%)
-	-	Nonanoic acid	12 (40%)
-	-	Oleic acid	12 (40%)
-	-	Palmitic acid	27 (90%)
-	-	Pentadecanoic acid or Hexadecanol	14 (46.7%)
-	-	Stearic acid	27 (90%)
-	Lipids	alpha-Monopalmitin	27 (90%)
-	-	beta-Monopalmitin	27 (90%)
-	-	beta-Monostearin	27 (90%)
-	-	Glycerol	26 (86.7%)
-	-	Thymol	15 (50%)
-	Organic acids	Pyruvic acid	20 (66.7%)
-	-	Succinic acid	7 (23.3%)
-	Other	Phosphoric acid	23 (76.7%)
-	-	Uridine	27 (90%)
Potential	Amino acids	Aspartic acid	3 (10%)
-	Benzene derivatives	Gentisic acid	4 (13.3%)
-		Phenol	2 (6.7%)
-	Carbohydrates	Fructose or similar ketohexose	1 (3.3%)
-	Fatty acids	Arachidic acid or 1-Heneicosanol	3 (10%)
-	-	Decanoic acid	1 (3.3%)
-	-	Lauric acid	4 (13.3%)
-	-	Methyl palmitate	2 (6.7%)
-	-	Methyl stearate	2 (6.7%)
-	Lipids	Gamma-Tocopherol	2 (6.7%)
-	Organic acids	Acetoacetate or 2-Aminoisobutanoic acid	3 (10%)
-	-	Glycolic acid	2 (6.7%)
-	-	Lactic acid	5 (16.7%)
-	-	Urea	2 (6.7%)
-	Other	1,2-Propanediol	1 (3.3%)
-	-	4-Hydroxypyridine or 3-Hydroxypyridine	3 (10%)
-	-	Ethanolamine	2 (6.7%)
-	-	O-Methylphosphate	3 (10%)
-	-	Prunetin or similar isoflavone	1 (3.3%)

**Table 2:** Distribution of adjusted R^2^ by analyte type. Known analytes had a significantly higher linearity than unknowns. Fisher’s exact test comparing known and unknowns *p*-value = 0.01.

Summary	No. (% )	Known	Unknown
R^2^_adj_ greater than 0.95	3	2 (1.6%)	1 (1.8%)
R^2^_adj_ (0.7, 0.95)	64	52 (41.3%)	12 (21.1%)
R^2^_adj_ (0.5, 0.7)	30	22 (29.7%)	8 (21.1%)
R^2^_adj_ less than 0.5	50	24 (32.5%)	24 (63.2%)

**Table 3:** Linear dynamic range for all analytes. The majority (90.5%) of analytes’ linear range was between 100 and 200 µL.

Plasma Extract Volume (µL)	No. Analytes (%)
75–100	8 (4.5%)
100–150	100 (55.9%)
150–200	62 (34.6%)
200–300	6 (3.4%)
300+	1 (0.6%)

**Table 4:** Experimental design for each batch in the limiting-dilution study. Eleven different plasma extract volumes repeated three times were included in each batch (total number of aliquots = 33). Each plasma extract volume was ballasted with 7.5:1 methanol/H_2_O (*v*/*v*) to bring the total volume to 700 µL. The entire limiting-dilution study consisted of 10 batches with identical experimental design.

Methanolic Plasma Extract Volume (µL)	Methanol/H_2_O Volume ^1^ (µL)	Equivalent Plasma Volume Injected ^2^ (nL)	Equivalent Plasma Concentration ^3^ (*v*/*v*)
0	700	0	0
25	675	5.7	1.25 × 10^−9^
50	650	11.3	2.49 × 10^−9^
75	625	17.0	3.74 × 10^−9^
100	600	22.6	4.98 × 10^−9^
150	550	33.9	7.48 × 10^−9^
200	500	45.2	9.97 × 10^−9^
300	400	67.9	1.50 × 10^−8^
400	300	90.5	1.99 × 10^−8^
600	100	135.7	2.99 × 10^−8^
700	0	158.4	3.49 × 10^−8^

^1^ 7.5:1 methanol/H_2_O (*v*/*v*) solution was used to bring the total volume up to 700 µL prior to drying. ^2^ Calculated using injection volume (5 μL out of 100 μL derivatized plasma) and split ratio (25:1): 0.1 mL of plasma × 106 nL/mL × (plasma extract volume/850) × (5/100)/26. ^3^ Calculated using weight-based estimate of total plasma volume 4.54 L.

**Table 5:** Recommendations for experimental design of GC-MS-based non-targeted profiling of human plasma metabolome, including recommendations on the inclusion of blanks, applications of linearity, control for repeatability and intermediate precision, establishment of linear range and treatment of unknowns.

Experimental Design	Recommendations
Establish method blanks	Include 3 blank samples in the beginning, middle and end of every sequence run
	Use both blanks and manual curation for contaminant profiling
	Establish a list of highly reproducible and potential contaminants
Linearity	Incorporate dilution into QC samples
	Metabolites showing linearity can be used as targets to validate the methodology and monitor changes
	Lack of linearity may indicate contaminant effect or saturation effect
Repeatability and intermediate precision	Batch should be included in reporting and analysis of non-targeted GC-MS profiling
Range	Linear dynamic range should be established through dilution studies
	Optimal concentration established through dilution studies should be used for metabolic profiling
Unknowns	Unknowns presenting as contaminants can be excluded from further analysis
	Highly-linear unknowns may be biologically important metabolites
	Reproducible, highly linear and non-contaminant unknowns should be added to the library or databases for future references

## Supplementary Materials

The following are available online at www.mdpi.com/2218-1989/7/3/45/s1, Figure S1: Example of positive (left) and negative (right) run order effect on contaminant levels. Contaminant on the left is an unknown with retention time 8.125 min; contaminant on the right is beta-monopalmitin, Table S1: Non-metabolite known contaminants detected in blanks, Table S2: Known analytes identified in the volunteer and NIST SRM 1950 plasma.
