# Supplementary material for: Recommendations for Improving Identification and Quantification in Non-Targeted, GC-MS-Based Metabolomic Profiling of Human Plasma
Source: Metabolites. 2017 Aug 25;7(3):45. doi: 10.3390/metabo7030045 (PMC5618330; doi:10.3390/metabo7030045)
Supplement: Supplementary file 1 [file metabolites-07-00045-s001.pdf]

Supplementary Materials: Methods for improved identification and quantification in GC-MS-based metabolomic profiling of human plasma

Hanghang Wang, Michael J. Muehlbauer, Sara K. O'Neal, Christopher B. Newgard, Elizabeth R. Hauser, James R. Bain, Svati H. Shah

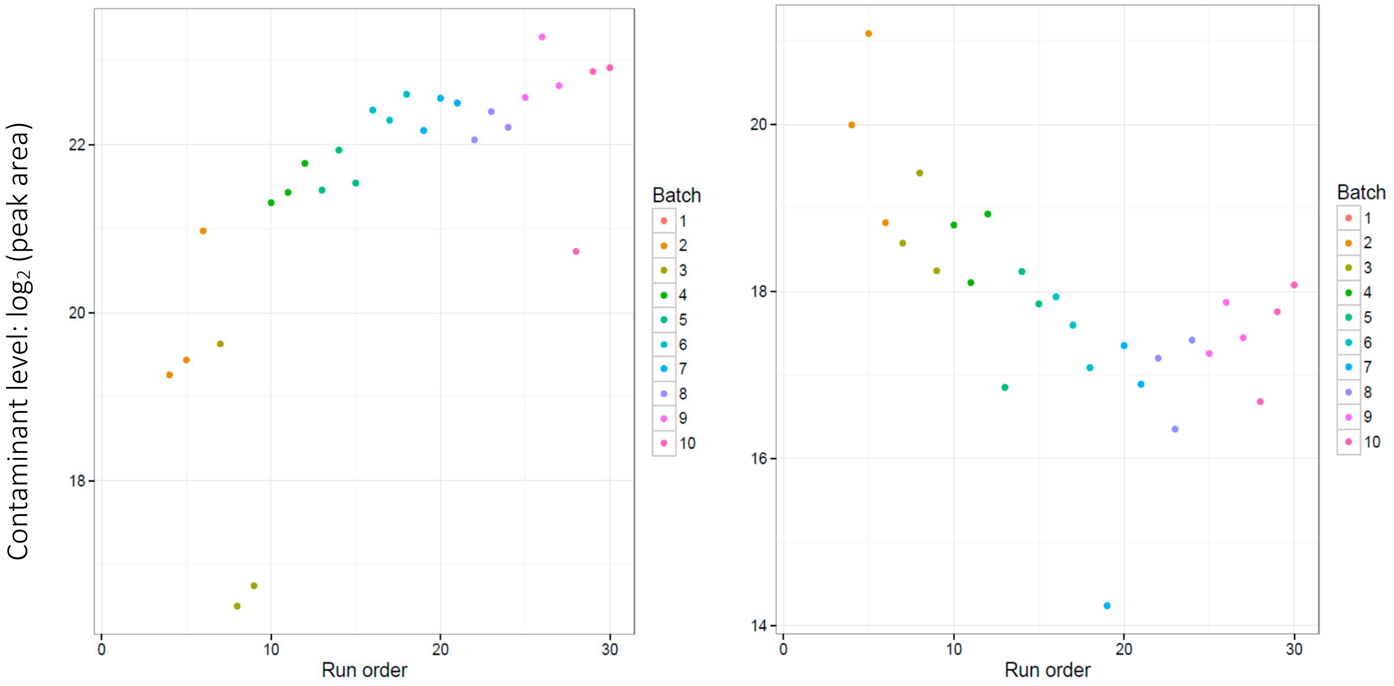

Figure S1. Example of positive (left) and negative (right) run order effect on contaminant levels. Contaminant on the left is an unknown with retention time 8.125 min; contaminant on the right is beta-monopalmitin.

Table S1. Non-metabolite known contaminants detected in blanks.

Type	Contaminant
**Alkane hydrocarbon**	C10 hydrocarbon
**Alkane hydrocarbon**	C11 hydrocarbon
**Alkane hydrocarbon**	C12 hydrocarbon
**Alkane hydrocarbon**	C13 hydrocarbon
**Alkane hydrocarbon**	C14 hydrocarbon
**Alkane hydrocarbon**	C15 hydrocarbon
**Alkane hydrocarbon**	C16 hydrocarbon
**Alkane hydrocarbon**	C17 hydrocarbon
**Alkane hydrocarbon**	C19 hydrocarbon
**Alkane hydrocarbon**	C20 hydrocarbon
**Alkane hydrocarbon**	C21 hydrocarbon

**Alkane hydrocarbon**	C23 hydrocarbon
**Alkane hydrocarbon**	C24 hydrocarbon
**Alkane hydrocarbon**	C25 hydrocarbon
**Silicone oil**	Eicosamethylcyclodecasiloxane
**Silicone oil**	Tetradecamethylcycloheptasiloxane
**Silicone oil**	Hexadecamethylcyclooctasiloxane
**Silicone oil**	Decamethylcyclopentasiloxane
**Silicone oil**	Tetracosamethylcyclododecasiloxane
**Silicone oil**	Triacontamethylcyclopentadecasiloxane
**Silicone oil**	Hexacosamethylcyclotridecasiloxane
**Silicone oil**	Dotriacontamethylcyclohexadecasiloxane
**Silicone oil**	Octacosamethylcyclotetradecasiloxane
**Silicone oil**	Dodecamethylpentasiloxane
**Silicone oil**	Tetratriacontamethylcycloheptadecasiloxane
**Silicone oil**	Eicosamethylcyclodecasiloxane
**Silicone oil**	Tetradecamethylcycloheptasiloxane
**Silicone oil**	Hexadecamethylcyclooctasiloxane
**Silicone oil**	Decamethylcyclopentasiloxane
**Silicone oil**	Tetracosamethylcyclododecasiloxane

Table S2. Known analytes identified in the volunteer and NIST SRM 1950 plasma.

CAS	Metabolite	Retention time	HMDB	Score***
57-55-6	1,2-Propanediol	5.8167	HMDB01881	81.3
127-17-3	Pyruvic acid	6.5404	HMDB00243	93.9
108-95-2	Phenol*	6.6788	HMDB00228	85.3
79-33-4	Lactic acid	6.7082	HMDB00190	96.8
594-61-6	2-Hydroxyisobutyric acid	6.7697	HMDB00729	82.5
79-14-1	Glycolic acid	6.9588	HMDB00115	81.7
56-41-7	Alanine	7.3753	HMDB00161	98.2
759-05-7	2-Ketovaline	7.4497	HMDB00019	87.2
565-70-8	2-Hydroxybutyric acid	7.721	HMDB00008	92.7
541-50-4	Acetoacetate	7.8322	HMDB00060	80.2
99-66-1	Valproic acid**	8.1218	HMDB01877	78.2
95-48-7	p-Cresol	8.1876	HMDB01858	77.3
626-64-2	4-Hydroxypyridine/3-Hydroxypyridine	8.2014	NA	90.9
150-83-4	beta-Hydroxybutyric acid	8.2333	HMDB00357	97.3
617-31-2	2-Hydroxyvaleric acid	8.2846	HMDB01863	90.2
100-61-8	N-Methylalanine	8.417	HMDB94692	94
812-00-0	O-Methylphosphate	8.4861	HMDB61711	82
816-66-0	2-Ketoleucine/ketoisoleucine	9.0049	HMDB00695	89.2
72-18-4	Valine	9.0587	HMDB00883	98.4
57-13-6	Urea	9.4046	HMDB00294	82.3
65-85-0	Benzoic acid	9.5694	HMDB01870	98.6

124-07-2	Octanoic acid	9.7836	HMDB00791	76.2
141-43-5	Ethanolamine	9.8078	HMDB00149	89.1
7664-38-2	Phosphoric acid	9.8367	HMDB02142	95.2
61-90-5	Leucine	9.8614	HMDB00687	91.7
56-81-5	Glycerol	9.873	HMDB00131	94.6
59-67-6	Nicotinic acid*	9.9251	HMDB01488	84.2
443-79-8	Isoleucine	10.1679	HMDB00172	97.5
147-85-3	Proline	10.248	HMDB00162	98.2
56-40-6	Glycine	10.3625	HMDB00123	98.2
110-15-6	Succinic acid	10.4553	HMDB00254	85.1
473-81-4	Glyceric acid	10.6504	HMDB00139	84.5
110-17-8	Fumaric acid	10.9615	HMDB00134	78.5
56-45-1	Serine	11.0719	HMDB00187	96.2
112-05-0	Nonanoic acid	11.1229	HMDB00847	82.1
535-75-1	Pipecolic acid	11.1796	HMDB00070	91
72-19-5	Threonine	11.4156	HMDB00167	97.9
107-95-9	beta-Alanine	12.002	HMDB00056	84.3
334-48-5	Decanoic acid	12.4007	HMDB00511	82.6
1068-84-4	Aminomalonic acid	12.557	HMDB01147	84.2
149-32-6	Erythritol	12.8735	HMDB02994	78.4
6968-16-7	Threitol	12.9749	HMDB04136	88.9
50-78-2	Acetylsalicylic acid/Salicylic acid	13.0476	HMDB01879	87.6
**CAS**	**Metabolite**	**Retention time**	**HMDB**	**Score**
63-68-3	Methionine	13.1541	HMDB00696	85.6
51-35-4	Hydroxyprolines	13.2323	HMDB00725	93.2
56-84-8	Aspartic acid	13.2791	HMDB00191	75
13752-84-6	Erythronic acid	13.4096	HMDB00613	80.8
60-27-5	Creatinine	13.5948	HMDB00562	89.7
3909-12-04	Threonic acid	13.607	HMDB00943	87.6
328-50-7	alpha Ketoglutaric acid	13.8106	HMDB00208	76.3
13095-48-2	2-Hydroxyglutaric acid	13.8287	HMDB00694	77.1
300-84-5	Hypotaurine	14.1839	HMDB00965	83
56-86-0	Glutamic acid	14.3533	HMDB00148	94.4
63-91-2	Phenylalanine	14.4664	HMDB00159	94.8
143-07-7	Lauric acid	14.7749	HMDB00638	79.6
147-81-9	Aldopentoses	14.8203	HMDB00646	83.1
70-47-3	Asparagine	14.9271	HMDB00168	92.5
542-32-5	2-Aminoadipic acid	15.3782	HMDB00510	78.2
488-81-3	Pentitols	15.4801	HMDB00508	83.9
10030-85-0	6-Deoxyhexose	15.5516	HMDB00849	76.8
585-84-2	Aconitic acid	15.8211	HMDB00958	77.9
34363-28-5	Glycerol 1-phosphate	15.9255	HMDB00126	84
56-85-9	Glutamine	16.0901	HMDB00641	94.7
1071-23-4	O-Phosphocolamine	16.1832	HMDB00224	76.1

820-11-1	3-Phosphoglyceric acid*	16.4257	HMDB00807	85.7
68-94-0	Hypoxanthine	16.4569	HMDB00157	83.9
5949-29-1	Citric acid/isocitric acid	16.5618	HMDB00094	94.6
70-26-8	Ornithine	16.5766	HMDB00214	93.3
495-69-2	Hippuric acid	16.8677	HMDB00714	77.6
154-58-5	1,5-Anhydroglucitol	16.9316	HMDB02712	91.1
58-08-2	Caffeine**	16.982	HMDB01847	85.5
87-81-0	Fructose or similar ketohexose	17.1104	HMDB03418	84.7
50-99-7	Glucose and other aldohexoses	17.4212	HMDB00122	97.4
5934-29-2	Histidine	17.6215	HMDB00177	80.3
56-87-1	Lysine	17.6675	HMDB00182	95.3
112-39-0	Methyl palmitate	17.7465	HMDB61859	92.9
14982-50-4	Hexuronic acid	17.7726	HMDB02545	81.3
60-18-4	Tyrosine	17.8357	HMDB00158	94.5
36653-82-4	Pentadecanoic acid	17.9434	HMDB03424	88.5
526-95-4	Gluconic acid or similar sugar acid	17.9886	HMDB00625	85.1
87-51-4	3-Indoleacetic acid	18.0846	HMDB00197	79.8
86879-39-2	CMPF	18.2267	HMDB61112	78.7
1190-94-9	5-Hydroxylysine*	18.5284	HMDB00450	78
373-49-9	Palmitoleic acid	18.7142	HMDB03229	84.3
57-10-3	Palmitic acid	18.9131	HMDB00220	99
87-89-8	Myoinositol	19.3116	HMDB00211	96.4

**CAS**	**Metabolite**	**Retention time**	**HMDB**	**Score**
69-93-2	Uric acid	19.3577	HMDB00289	97.3
112-63-0	Methyl linoleate	19.3946	HMDB34381	83.6
112-62-9	Methyl oleate	19.455	NA	85.9
112-61-8	Methyl stearate	19.6902	HMDB34154	88.7
506-12-7	Heptadecanoic acid	19.8346	HMDB02259	87.4
1821-52-9	3-Indolelactic acid	20.0786	HMDB00671	77.3
60-33-3	Linoleic acid	20.4453	HMDB00673	93.1
73-22-3	Tryptophan	20.4619	HMDB00929	93.7
112-80-1	Oleic acid	20.4979	HMDB00207	94.9
57-11-4	Stearic acid	20.7263	HMDB00827	99
56-89-3	Cystine	21.1349	HMDB00192	82
1445-07-4	Pseudouridine	21.439	HMDB00767	80.7
506-32-1	Arachidonic acid (also Eicosapentaenoic acid)	21.8055	HMDB01043	83.4
103529-92-6	Myoinositol-2-phosphate	22.0623	HMDB00213	83.4
506-30-9	Arachidic acid	22.3977	HMDB02212	80
58-96-8	Uridine	22.4234	HMDB00296	78.2
50-67-9	Serotonin	22.5423	HMDB00259	84.8
23470-00-0	beta-Monopalmitin	23.2646	HMDB11533	88.4
6217-54-5	Docosahexaenoic acid	23.3277	HMDB02183	84.1
542-44-9	alpha-Monopalmitin	23.5287	HMDB05356	97.6
112-85-6	Docosanoic acid	23.8737	HMDB00944	80.1

57-50-1	Sucrose and similar disaccharides	23.8868	HMDB00258	79
69-79-4	Maltose or similar disaccharide	24.6515	HMDB00163	75.7
31566-31-1	beta-Monostearin	24.71	HMDB11535	93.4
123-94-4	alpha-Monostearin	24.9697	HMDB31075	99
552-59-0	Prunetin or similar isoflavone*	25.5472	HMDB34127	86.5
54-28-4	gamma-Tocopherol	26.5188	HMDB01492	77.8
10191-41-0	alpha-Tocopherol	27.4657	HMDB01893	92
57-88-5	Cholesterol	27.6039	HMDB00067	98.2
474-62-4	Campesterol*	28.2744	HMDB02869	92.2
19044-06-5	beta-Sitosterol	28.9001	HMDB00852	94
1109-28-0	Trisaccharide*	30.1518	HMDB01262	84.7

* Analytes detected in volunteer plasma only.
** Analytes detected in NIST SRM 1950 standard plasma only.
*** Reverse score by matching to the library.
